# Role of adenylyl cyclase in reduced β-adrenoceptor-mediated vasorelaxation during maturation

**DOI:** 10.1590/1414-431X20165285

**Published:** 2016-07-04

**Authors:** O.A. López-Canales, M.C. Castillo-Hernandez, H. Vargas-Robles, A. Rios, J.S. López-Canales, B. Escalante

**Affiliations:** 1 Pharmacology Department, Superior School of Medicine, Polytechnic National Institute, Mexico City, Mexico; 2 Molecular Biomedicine Department, Center of Research and Advanced Studies, Unidad Zacatenco, Mexico City, Mexico; 3 Center of Research and Advanced Studies, Monterrey, Nuevo León, Mexico; 4 Perinatology National Institute "Isidro Espinosa de los Reyes", Mexico City, Mexico

**Keywords:** β adrenergic receptors, Vascular tissue, Adenyl cyclase

## Abstract

Beta-adrenergic receptor (βAR)-dependent blood vessel relaxation is impaired in older animals and G protein activation has been suggested as the causative mechanism. Here, we investigated the role of βAR subtypes (β1AR, β2AR, and β3AR) and cAMP in maturation-dependent vasorelaxation impairment. Aortic rings from 15 Sprague-Dawley male rats (3 or 9 weeks old) were harvested and left intact or denuded of the endothelium. Vascular relaxation in aortic rings from younger and older groups was compared in the presence of βAR subtype agonists and antagonists along with cAMP and cGMP antagonists. Isolated aortic rings were used to evaluate relaxation responses, protein expression was evaluated by western blot or real time PCR, and metabolites were measured by ELISA. Expression of βAR subtypes and adenylyl cyclase was assessed, and cAMP activity was measured in vascular tissue from both groups. Isoproterenol- and BRL744-dependent relaxation in aortic rings with and without endothelium from 9-week-old rats was impaired compared with younger rats. The β1AR antagonist CGP20712A (10^-7^ M) did not affect isoproterenol or BRL744-dependent relaxation in arteries from either group. The β2AR antagonist ICI-118,551 (10^-7^ M) inhibited isoproterenol-dependent aortic relaxation in both groups. The β3AR antagonist SR59230A (10^-7^ M) inhibited isoproterenol- and BRL744-dependent aortic ring relaxation in younger but not in older rats. All βAR subtypes were expressed in both groups, although β3AR expression was lower in the older group. Adenylyl cyclase (SQ 22536) or protein kinase A (H89) inhibitors prevented isoproterenol-induced relaxation in younger but not in older rats. Production of cAMP was reduced in the older group. Adenylyl cyclase III and RyR3 protein expression was higher in the younger group. In conclusion, altered expression of β3AR and adenylyl cyclase III may be responsible for reduced cAMP production in the older group.

## Introduction

Blood vessel relaxation induced by the catecholamine system is mediated through β adrenergic receptors (βAR). Although vascular βAR were originally classified as beta 2 (β2AR) and beta 1 (β1AR) adrenergic receptor subtypes, later studies described atypical β adrenergic receptors that included β3AR (1). Further characterization described β3AR as a relevant mediator in catecholamine-induced vasodilation (2-4). Catecholamine-dependent blood vessel relaxation is impaired in older animals (5). In humans, loss of vascular relaxation in elderly people could contribute to clinical development of hypertension or cardiovascular diseases, as orthostatic hypotension and arterial insufficiency are present in older populations (6). Several authors have explored the mechanism involved in βAR-dependent impaired vascular relaxation. Initial reports suggested decreased cAMP production and phosphodiesterases as the mechanism involved in the adrenergic vasorelaxation impairment (5). Later on, decreased function of the stimulatory GTP-binding protein (Gαs) was associated with β2AR-induced vasodilation impairment in aortas from aged rats (7). This idea was further explored by several authors that either supported decreased (8) or increased (9) Gαs protein expression as the mechanism for age-dependent adrenergic vasorelaxation impairment. More recently, a differential activation of Gα protein has been suggested as an explanation for this controversy: activation of Gαs decreased, whereas Gαi increased in older animals (10).

Most of the studies on age-related βAR-mediated vascular relaxation have focused on β1AR or β2AR, but few studies have evaluated the role of β3AR (1,4). However, it has been suggested that the three βAR subtypes work in concert to render adrenergic vascular relaxation. All βAR receptors couple to Gαs protein and elicit cAMP production (11). Under normal stimulation, β2AR- and β1AR-dependent cAMP production is modulated by PKA phosphorylation, leading to reduced cAMP production and reduced β2AR- and β1AR-mediated vasorelaxation (12). However, β3AR does not require this PKA phosphorylation mechanism, which allows a prolonged vasorelaxation effect compared with that induced by β2AR and β1AR (13). These combined results suggest that age-dependent changes in adrenergic vasorelaxation could be associated with differential expression of β-adrenergic receptor second messengers.

Understanding the causes of age-related decline in βAR-mediated vasodilation-signaling may help identify a therapeutic target for treating cardiovascular diseases. In the present study, we investigated βAR subtypes and cAMP-dependent vasorelaxation in maturation-dependent adrenergic vasorelaxation. We evaluated the mechanisms of βAR-mediated cAMP signaling by testing vasorelaxation and cAMP activity in aortic rings from 3- and 9-week-old rats in the presence of specific β1AR, β2AR, β3AR, and cAMP antagonists. We also evaluated protein content and mRNA expression of βAR in thoracic aorta.

## Material and Methods

Fifteen male Sprague-Dawley rats (3 or 9 weeks old) were used throughout the experiments. Rats were fed with a balanced diet and had water *ad libitum*. All procedures conformed to the National Institutes of Health "Guide for the Care and Use of Laboratory Animals" (1996) and were approved by the Institutional Ethics Review Committee for Animal Experimentation of the Center of Research and Advanced Studies, IPN (approval No. 479-10).

### Preparation of rat aortic rings

Rats were sacrificed by cervical dislocation. The thoracic aorta was carefully removed, and placed directly into ice-cold Krebs-Henseleit bicarbonate solution (117.8 mM NaCl, 6.0 mM KCl, 1.6 mM CaCl_2_, 1.2 mM MgSO_4_, 1.2 mM KH_2_PO_4_, 24.2 mM NaHCO_3_, 11.0 mM glucose, 0.027 mM EDTA), equilibrated with 95% O_2_ and 5% CO_2_, pH 7.4. Then, periadventitial fat was removed. Care was taken during the complete procedure to ensure the integrity of the vascular endothelium. For experiments with endothelium-denuded aortic rings, the endothelium was removed by gently rubbing the intimal surface of the vessels. Aortic rings (3-4 mm wide) were mounted in 5 mL water-jacketed organ baths at 37°C and equilibrated for 1.5 to 2.0 h. A maximum of two aortic rings from either 3- or 9-week-old rats were used simultaneously. Basal tone was set at 1.5 and 2.0 g, respectively. Changes in tension were measured using a pressure transducer (TSD125) connected to a computerized data acquisition system (MP150-BIOPAC, BIOPAC Systems, USA). This procedure was determined to produce optimal conditions for reproducible isometric force development.

### Experimental procedure

The presence of endothelium was confirmed by assessing the effectiveness of acetylcholine (ACh, 10^-6^ M) to relaxed aortic rings pretreated with phenylephrine (PE, 10^-6^ M). The endothelium denudation was confirmed by the incapability of aortic rings to relax in response to ACh (10^-6^ M). Concentration-response curves for ACh, isoproterenol, dobutamine, salbutamol, BRL3744, and sodium nitroprusside were obtained in aortic rings pretreated with PE by cumulative addition of the drug to the organ bath. The concentration was increased only after maximal response to the previous concentration was attained. Specific inhibitors and antagonists were used as follows: βAR antagonists, 10^-7^ M CGP20712A for β1AR, 10^-7^ M ICI-118,551 for β2AR, and 10^-7^ M SR59230A for β3AR; adenylyl cyclase inhibitor, 10^-7^ M SQ22536; PKA inhibitor, 10^-7^ M H89; guanylyl cyclase inhibitor, 10^-7^ M ODQ; and PGK inhibitor, 10^-7^ M K75823. The isoproterenol concentration-response curve or specific agonist curve were determined, then aortic rings were incubated with the inhibitor or antagonist for 30 min, and the isoproterenol concentration-response curves were repeated. Vascular relaxation is reported as percentage, considering basal tension before PE stimulus as 100% vascular relaxation and PE-induced tension as 0% vascular relaxation.

### Western blot

Frozen aortas were ground to powder in a mortar and homogenized with ice-cold lysis buffer (50 mM Tris-HCl pH 7.5, 2.5 mM EDTA, 137 mM NaCl, 1% NP40, 5%glycerol, 1.5 μg/mL leupeptin, 1.0 μg/mL aprotinin, and phenylmethanesulfonyl fluoride (PMSF). Homogenates were centrifuged at 10,000 *g* for 10 min at 25°C, supernatant was collected, and protein was measured by Bradford's method. Then, 100 μg of protein was mixed with loading buffer (50 mM Tris- HCl, pH 6.5, 2% SDS, 10% glycerol, 0.02% bromophenol blue and heated at 100°C for 2 min. Protein was detected on 2% SDS/PAGE gels under reducing conditions, and then transferred to Hybond-P PVDF membranes (Amersham, GE Healthcare, UK). Blots were blocked for 40 min with TBS containing 5% skim dry milk and 0.5% Tween 20. Immunoblot analysis was performed with the following antibodies (Santa Cruz Biotechnology, USA): β1-AR (sc-568, 1:100), β2-AR (sc-9042, 1:250) (14), anti-β3-AR (sc-1473, 1:500) (15 [Bibr B16] [Bibr B17] [Bibr B18]) and anti-_p_β2AR (Ser 355/356, sc-16719, 1:200). Anti-actin antibody (A2066, 1:2000; Sigma-Aldrich, USA) was used as loading control. All antibodies were diluted in blocking solution, and blots were incubated overnight at 4°C. Blots were then washed three times with TBS containing 0.5% Tween 20 and incubated with the corresponding horseradish peroxidase-conjugated secondary antibody. Immunoreactive bands were detected by enhanced chemiluminescence (Amersham, GE Healthcare) using Kodak BioMax ML film, and analyzed with 1D image analysis software (Kodak, USA). Values for each band are expressed in arbitrary units (AU). All samples from each βAR (5 aortas from each age group) were run simultaneously to eliminate intra-assay variation. Blots presented in figures represent one of the five different experiments. The βAR/actin densitometry ratios were calculated for each group and are reported as means±SE.

### Gene expression analysis

Aortas from 3- and 9-week-old rats were homogenized and total RNA was extracted using TRIzol (Life Technologies, USA). RNA integrity was checked in agarose gels, and 1.0 µg RNA was used for reverse transcriptase reactions. Gene expression analysis was performed using the FastStart SYBR Green Master (Rox) kit (Roche Applied Science, USA) and a 7500 Real Time Thermal Cycler (Applied Biosystems, USA). Specific primers for adenylyl cyclase subtypes and the calcium-related protein RyR3 target genes are shown in Table 1.

**Table:**
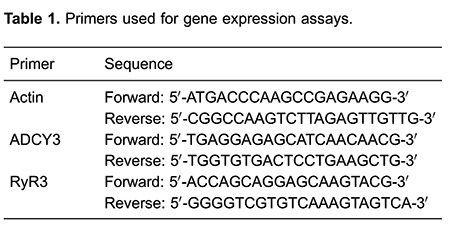

Relative gene expression was normalized to the constitutive expression of *α-actin*. Gene expression values were determined using the 2^-ΔΔCT^ formula. Data in figures represent 1 of the 5 different experiments. AR/actin densitometry value ratios were calculated for each group and reported as means±SE.

### cAMP enzyme immunoassay

Aortic rings were incubated in 500 µL of Krebs solution alone or with cAMP-specific phosphodiesterase inhibitor (10^-7^ M rolipram) at 37°C in a shaker bath. cAMP stimulation was initiated by adding 10^-5^ M isoproterenol for 15 min. Samples were rapidly frozen after collection, and intracellular cAMP levels were determined using a cyclic AMP EIA kit (Cayman Chemical, USA) according to the manufacturer's instructions.

### Statistical analysis

Concentration-response curves were compared using two-way analysis of variance (ANOVA) and specific differences between concentrations and rats were compared by modified Bonferroni *t*-test. Comparisons between two or more groups were performed by one-way ANOVA followed by Newman Keuls test to identify specific differences. Statistical analyses were performed using GraphPad Software Prism 5 for Mac OS X version 5.0 (USA). Data are reported as means±SE and P<0.05 was considered statistically significant.

## Results

### Isoproterenol-dependent vascular relaxation

**Figure 1:**
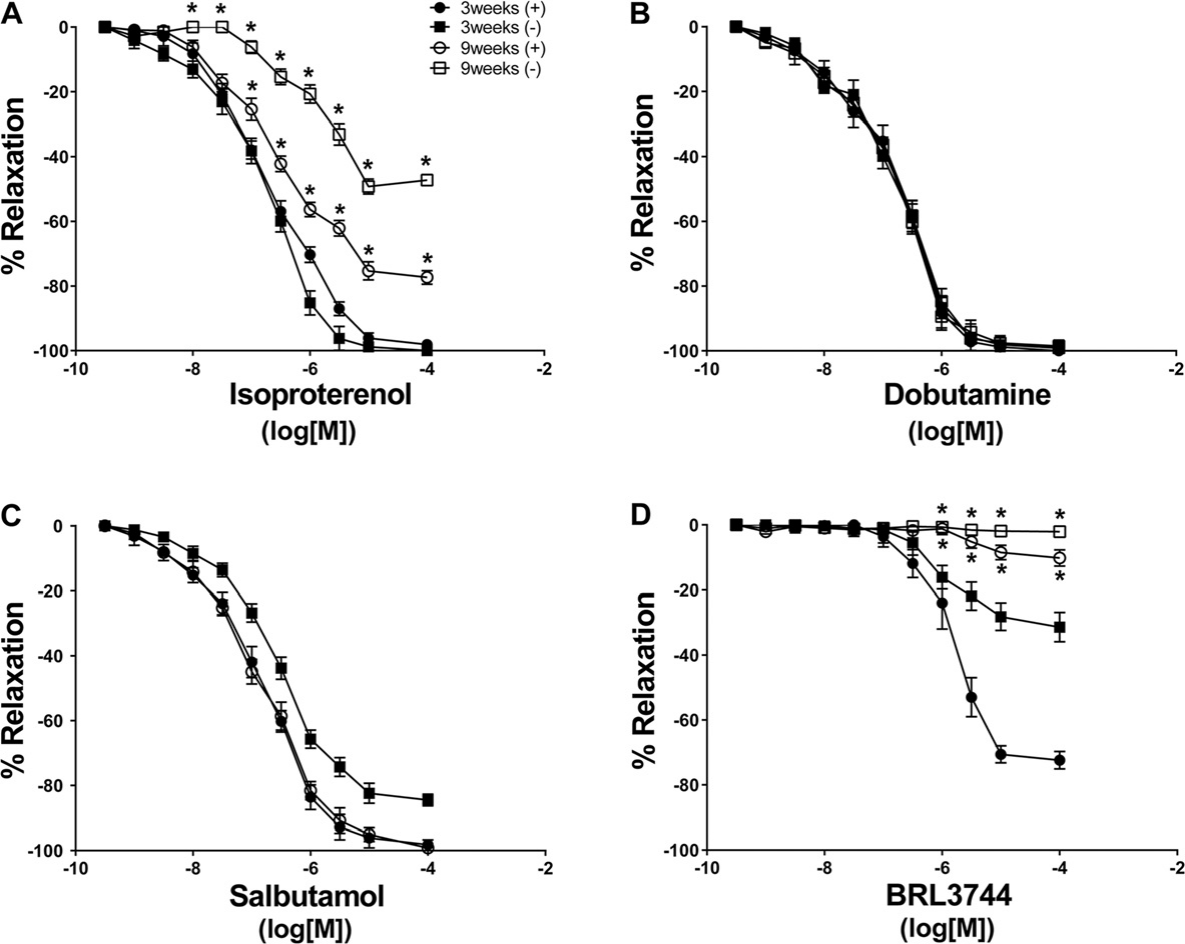
Concentration response curves in precontracted (treated with phenylephrine) aortic rings. The relaxant response to isoproterenol (*A*), dobutamine (*B*), salbutamol (*C*), and BRL3744 (*D*) was evaluated in endothelium-intact aortic rings (+) and endothelium-denuded aortic rings (−) of 3- and 9-week-old rats. Data are reported as the mean±SE of 5 different animals. *P<0.05 *vs* 3-week-old (ANOVA followed by modified Newman Keuls *t*-test).

PE produced similar dose response-dependent contractions in endothelium-intact aortic rings from 3- and 9-week-old rats. Maximal contractions were 1.22±0.13 and 1.49±0.25 g, respectively. In endothelium-denuded rings from 3- and 9-week-old rats, maximal contractions elicited by PE were 1.32±0.12 and 1.67±0.15 g, respectively. Isoproterenol on precontracted (PE treated) aortic rings from 3- and 9-week-old rats produced a concentration-dependent relaxation in vessels from both groups (Figure 1 and Table 2). However, isoproterenol-induced vascular relaxation was lower in aortic rings from 9-week-old rats than in 3-week-old rats (Figure 1A). This impairment was larger in endothelium-denuded aortic rings than in aortic rings with intact endothelium. Maximal % of isoproterenol-induced relaxation was 100±0% compared with 77.3±2.1% in endothelium-intact aortic rings from 3- and 9-week-old rats, respectively. Whereas, isoproterenol-induced relaxation was 98.1±1.5% compared with 47.2±1.7% in endothelium-denuded aortic rings from 3- and 9-week-old rats, respectively (Figure 1A). No difference in aortic ring relaxation was observed with acetylcholine or sodium nitroprusside agonists treatment in either group.

**Table:**
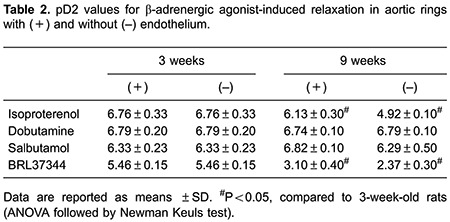

### Relaxation response to adrenergic agonists in older rats

To identify the possible adrenergic receptor involved in impaired adrenergic relaxation in 9-week-old rats, we tested different β-adrenergic agonists (Table 2). Dobutamine (β1 agonist, Figure 1B) and salbutamol (β2 agonist, Figure 1C) induced dose-dependent aortic ring relaxation in both younger and older rats. There were no differences in relaxation responses between 3- and 9-week-old rats in the presence or absence of endothelium. BRL3744 (β3 agonist) induced concentration-dependent vascular relaxation in both groups; however, BRL3744-mediated aortic ring relaxation was lower in 9-week-old rats compared with that of younger rats (Figure 1D). BRL3744-mediated impairment was more severe in endothelium-denuded aortic rings than in endothelium-intact aortic rings. Maximal relaxation induced by BRL3744 was 31.4±4.5 and 2.1±0.6% in endothelium-denuded aortic rings of 3- and 9-week-old rats, respectively, compared with 72±2.7 and 10.2±2.5% in endothelium-intact aortic rings of 3- and 9-week-old rats, respectively (Figure 1D).

### Relaxation response to adrenergic antagonists in older rats

To further explore the β-adrenergic subtypes involved in vascular relaxation impairment in older rats, we tested the effects of βAR agonists in the presence of βAR antagonists. The results are presented as pD2 values in Table 3. The presence of β1AR antagonist CGP20712A inhibited dobutamine-induced relaxation, but did not affect salbutamol- or BRL3744-induced responses. The addition of β2AR antagonist ICI-118,551 inhibited isoproterenol- and salbutamol-induced relaxation, but did not affect the dobutamine-induced response. The presence of β3AR antagonist SR59230A inhibited BRL3744-induced relaxation, but did not affect dobutamine- and salbutamol-induced responses.

**Table:**
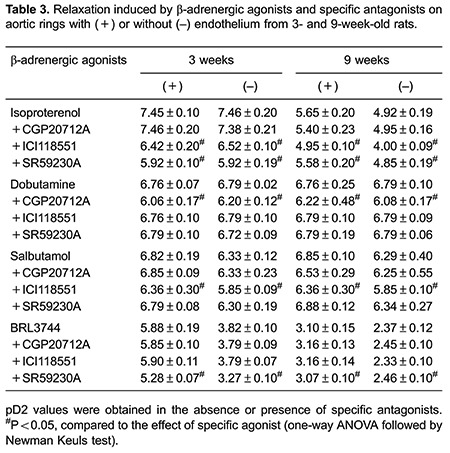

**Figure 2:**
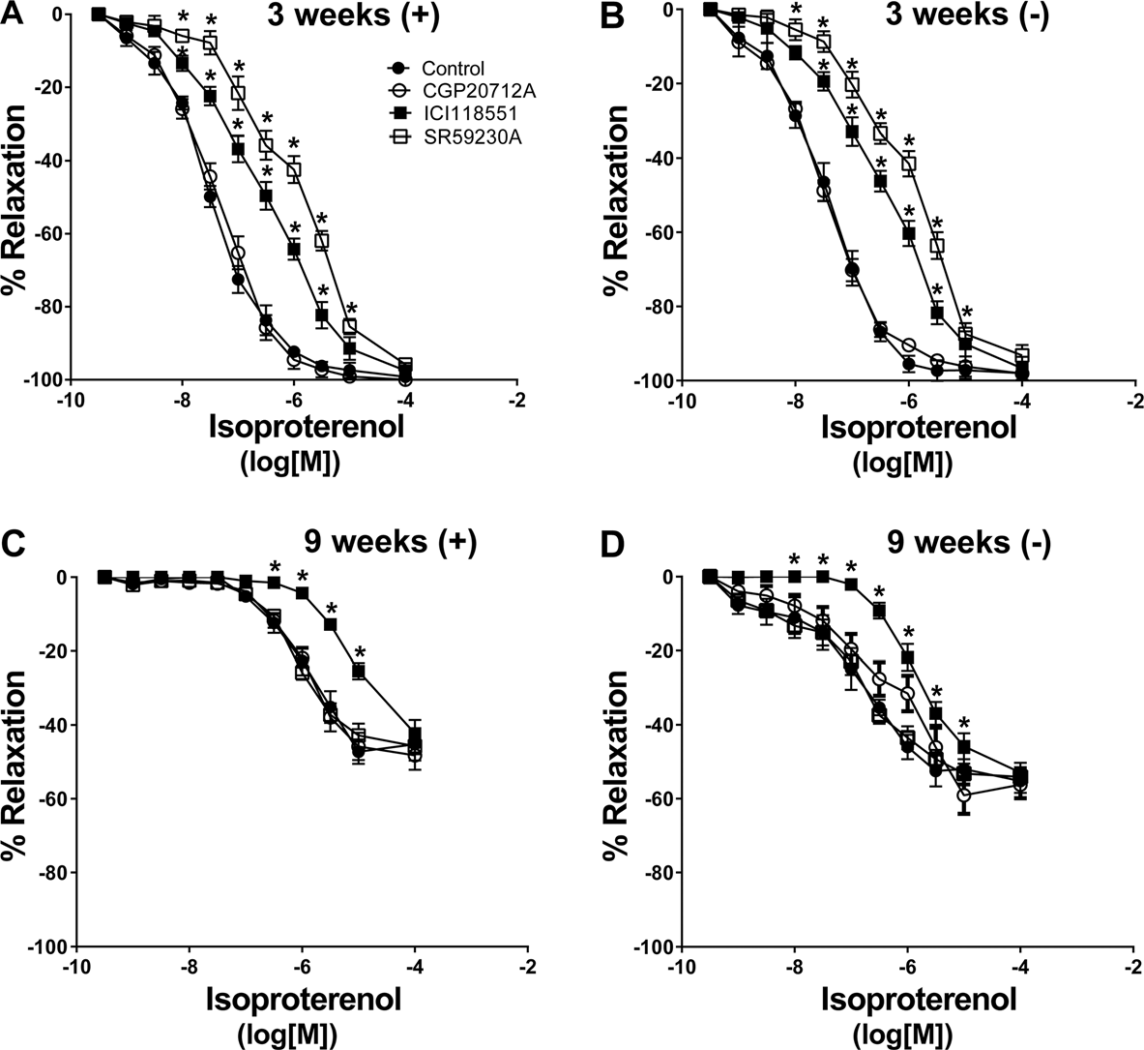
Concentration response curves in precontracted (treated with phenylephrine) aortic rings. The relaxant response to isoproterenol was evaluated in endothelium-intact aortic rings (+) and endothelium-denuded aortic rings (−) of 3- and 9-week-old rats in the absence or presence of the β1 receptor antagonist CGP20712A (10^-7^ M), β2 receptor antagonist ICI118551 (10^-7^ M), and β3 receptor antagonist SR59230A (10^-7^ M). Data are reported as the mean±SE of 5 different animals. *P<0.05 *vs* control (ANOVA followed by modified Newman Keuls *t*-test).

**Figure 3:**
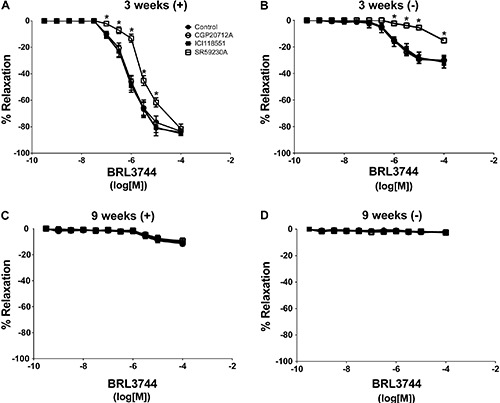
Concentration response curves in precontracted (treated with phenylephrine) aortic rings. The relaxant response to BRL3744 was evaluated in endothelium-intact aortic rings (+) and endothelium-denuded aortic rings (−) (*A* and *C*) of 3- and 9-week-old rats (*B* and *D*) in the absence or presence of the β1 receptor antagonist CGP20712A (10^-7^ M), β2 receptor antagonist ICI118551 (10^-7^ M), and β3 receptor antagonist SR59230A (10^-7^ M). Data are reported as the mean±SE of 5 different animals. *P<0.05 *vs* control (ANOVA followed by modified Newman Keuls *t*-test).

These antagonists were tested on isoproterenol- and BRL3744-induced relaxation in aortic rings with and without endothelium. Isoproterenol-induced relaxation was tested in presence of specific βAR antagonists (Figure 2). The presence of β1AR antagonist CGP20712A did not affect isoproterenol-dependent aortic ring relaxation in younger and older rats. Addition of the β2AR antagonist ICI-118,551 inhibited isoproterenol-dependent aortic ring relaxation in both groups (Figure 2A-D). The β3AR antagonist SR59230A inhibited isoproterenol-dependent aortic ring relaxation in 3-week-old rats but not in 9-week-old rats. BRL3744-dependent vascular relaxation was not affected by the β1AR antagonist CGP20712A or the β2AR antagonist ICI-118,551 in either group. However, the β3AR antagonist SR59230A inhibited BRL3744-dependent vascular relaxation in aortic rings of 3-week-old rats, but not in those of 9-week-old rats (Figure 3A-D).

### Differential protein expression of β1AR, β2AR, and β3AR

**Figure 4:**
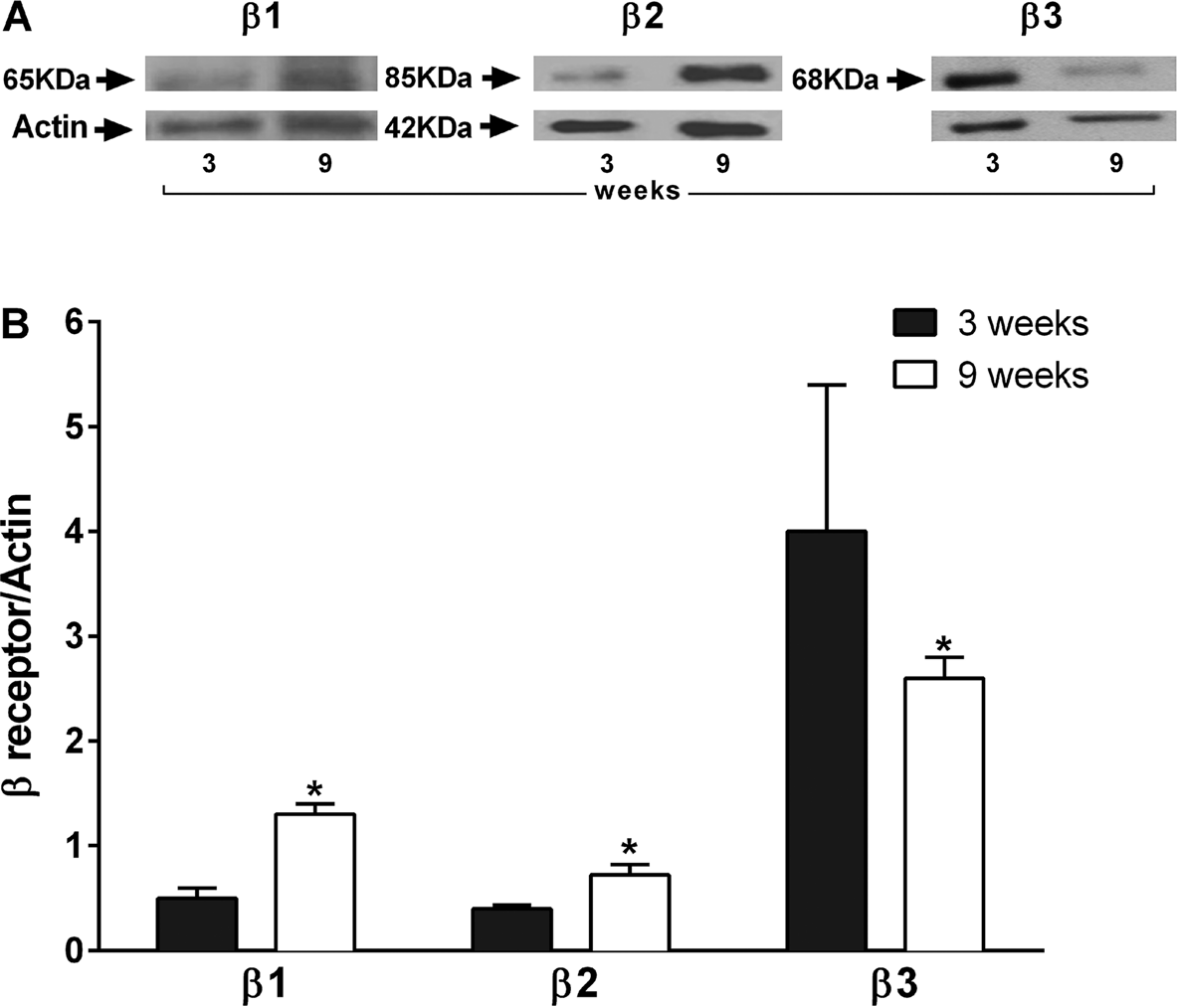
Comparative analysis of β-adrenergic receptor protein expression in aortic tissue of 3- and 9-week-old rats. The western blot is representative of 5 different experiments for each specific β receptor, with actin used as control (*A*). Graph represents the β-adrenergic receptor/actin ratio of 3- and 9-week-old rats (*B*). Data are reported as the mean±SE of 5 different rats. *P<0.05 *vs* 3-week-old rats (one-way ANOVA followed by Newman Keuls test).

**Figure 5:**
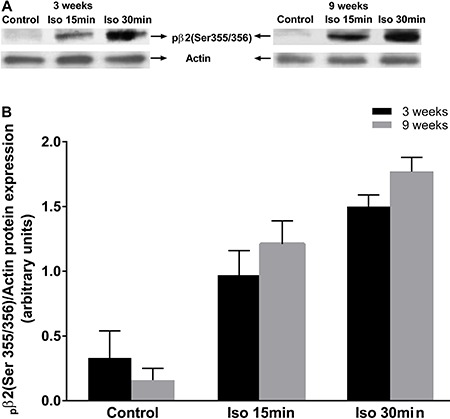
Comparative analysis of β-adrenergic receptor protein phosphorylation in aortic tissue of 3- and 9-week-old rats. Blots are representative of five different experiments, with actin as control (*A*). β-adrenergic receptor phosphorylation was evaluated in the absence of receptor agonist (control) or in the presence of isoproterenol (Iso, 10^-5^ M). Aortic tissue of 3- and 9-week-old rats was analyzed 15 or 30 min after addition of Iso. Graph represents the _p_β2/actin ratio (*B*). Data are reported as the mean±SE of 5 different rats.

Higher levels of β1AR and β2AR protein expression were detected in vascular tissue of 9-week-old rats compared with those of 3-week-old rats). The protein expression levels of β3AR were lower in vascular tissue of 9-week-old rats compared with that in 3-week-old rats (Figure 4A and B). No differences were observed in β2AR receptor phosphorylation under basal conditions or after isoproterenol stimulation in vascular tissue of younger and older rats (Figure 5A and B).

### Differential response to adenylyl cyclase pathway inhibitors

**Figure 6:**
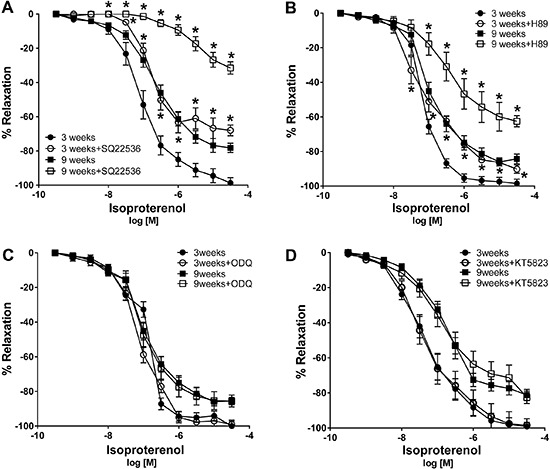
Concentration response curves in precontracted (treated with phenylephrine) aortic rings. The relaxant response to isoproterenol was evaluated in aortic rings of 3- and 9-week-old rats in the absence or presence of adenylyl cyclase inhibitor SQ22536 (10^-7^ M) (*A*), protein kinase A inhibitor H89 (10^-7^ M) (*B*), guanylyl cyclase inhibitor ODQ (10^-7^ M) (*C*), and protein kinase G inhibitor KT5823 (*D*) (10^-7^ M). Data are reported as the mean±SE of 5 different animals. *P<0.05 *vs* control (ANOVA followed by modified Newman Keuls *t*-test).

The adenylyl cyclase inhibitor SQ22536 and the protein kinase A inhibitor H89 significantly inhibited isoproterenol-induced vascular relaxation in aortic rings of 3-week-old rats but not in those of 9-week-old rats (Figure 6A and B). The guanylyl cyclase inhibitor ODQ and the PGK inhibitor KT75823 did not affect isoproterenol-induced relaxation in aortic rings of younger and older rats (Figure 6C and D).

cAMP production by aortic rings was measured after stimulation with isoproterenol. The results indicated that cAMP production decreased in aortic rings of 9-week-old rats (3.3±1 pmol/mL) compared with that in 3-week-old rats (20±2 pmol/mL). Similar results were observed in the presence of the phosphodiesterase inhibitor rolipram (8±1 and 26±3 pmol/mL for 9- and 3-week-old rats). However, dose response curves of forskolin-induced vascular relaxation showed no differences between aortic rings from younger and older rats. Thus, pD2 values were 6.9±0.13 and 6.6±0.2, and cAMP production was 20±2 and 25±5 pmol/mL for 3- and 9-week-old rats, respectively.

**Figure 7:**
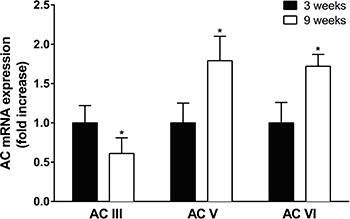
Comparative analysis of transcript abundance of adenylyl cyclase (AC) isoforms. qPCR was performed on cDNA from 3- and 9-week-old rats. The relative expression of AC III, V, or VI was calculated by the 2^-ΔΔCT^ method and normalized with respect to the mean value of 3-week-old controls. All values were normalized. Data are reported as the mean±SE of 5 different animals. *P<0.05 *vs* 3-week-old rats (one-way ANOVA followed by Newman Keuls test).

The expression of genes encoding adenylyl cyclase III, V, and VI showed increased expression of type V and VI and decreased expression of type III in vascular tissue of 9-week-old rats compared with that in 3-week-old rats (Figure 7). The calcium-related protein RyR3 mRNA transcript levels increased 2.5±0.4 fold in vascular tissue of 9-week-old rats compared with that in 3-week-old rats.

## Discussion

In the present study, we demonstrated that vascular relaxation impairment is associated with maturation, and we suggest that changes in expression of genes encoding β3AR and adenylyl cyclases are responsible for the altered vascular function. Our observation that vasorelaxation impairment induced by ACh or sodium nitroprusside did not change in 9-week-old rats compared with 3-week-old rats supports a specific role for βAR in maturation-dependent vasorelaxation impairment, as described in previous studies (7). Reduced βAR-induced vasorelaxation associated with aging has been reported in several studies (6,8,), similarly to our results. However, the age of animals in those reports ranged from 6- to 24-month-old compared with 9-week-old rats used in the present study, suggesting that this effect is related to maturation rather than with aged animals.

The presence of several βAR subtypes in rat aortic rings was demonstrated by the use of specific receptor agonists and antagonists and further supported by western blot. This suggests that all 3 adrenergic receptor subtypes are involved in the isoproterenol induced vasorelaxation, as reported previously (19). Beta adrener-gic-dependent vasorelaxation has been described as endothelium-dependent and endothelium-independentin rat aorta (20,21). Our data demonstrated that endothelium removal decreased salbutamol and BRL37344- induced vasorelaxation but did not affect dobutamine-induced vasorelaxation (Table 2). Thus, we suggest that adrenergic-mediated vasorelaxation of the endothelium is related to the receptor involved.

Identification of the βAR involved in maturation-dependent vasorelaxation impairment was suggested by the decreased vasorelaxation in 9-week-old rats induced by specific β2 and β3 agonists, and supported by the absence of inhibitory effect of specific antagonists. Moreover, decreased expression of the β3 receptor further supports the role of this receptor in the impaired vasorelaxation.

Relaxation impairment is more evident if endothelium has been removed (53±3% in endothelium-denuded arteries) compared with endothelium-intact rings (33±4%). This result suggests an important change of the adrenergic signaling pathway in smooth muscle cells. This is in agreement with a report showing reduction in isoproterenol-induced cAMP accumulation in cultured smooth muscle cells obtained from older rats (22).

Because no differences in β2 adrenergic receptor phosphorylation were observed in younger and older rats, we suggest that vasorelaxation impairment in 9-week-old rats was not associated with changes in receptor activation.

Adenylyl cyclase stimulation with cAMP production is the classical AR signaling pathway in vascular smooth muscle (23). Therefore, we explored cAMP pathway as being responsible for the age-maturation impairment. In older rats, there was a reduction of cAMP-dependent β3 vasorelaxation, which was evidenced by the fact that neither adenylyl cyclase inhibitors nor PKA inhibitors affected isoproterenol-induced relaxation in aortic rings from 9-week-old rats. cAMP production was reduced in aortic rings from 9-week-old rats, which further supports the hypothesis that the isoproterenol effect was no longer cAMP-dependent in older rats. Reduced cAMP production can be explained by reduced initial stimulus, reduced adenylyl cyclase activation, or elevated phosphodiesterase activation. Forskolin-induced adenylyl cyclase activity did not differ in aortic ring relaxation in younger and older rats suggesting that cAMP activation is not the mechanism involved in the age-maturation cAMP reduction. However, adenylyl cyclase III expression was reduced in vascular tissue of 9-week-old rats, suggesting that reduced cAMP levels may result from a change in adenylyl cyclase subtype that interacts with β-receptors. The calcium receptor RyR3 expression levels increased in 9-week-old rats. Adenylyl cyclase calcium-dependent regulation has been associated with adenylyl cyclase inhibition rather than phosphodiesterase activation (24). Cytoplasmic calcium levels are reported to regulate adenylyl cyclase activity in vascular smooth muscle cells (25,26). Therefore, our data suggest that reduced cAMP production in 9-week-old rats was associated with increased calcium levels and to increased RyR3 receptor, which inhibits adenylyl cyclase III isoform in aortic rings.

The present results indicate that impaired βAR relaxation may be associated with age maturation. Our results showed evidence of this process at the onset of sexual maturation. Therefore, we suggest that increased male sexual hormones promote changes in the AR signaling pathway as part of physiological blood vessel maturation. Impaired βAR relaxation present in adult or older rats may be associated with blood vessel damage. Thus, the role of testosterone related to changes in vascular response requires further investigation.
